# Discerning clinical target volume of endometrial cancer via a lightweight multi-phase delineation framework

**DOI:** 10.1186/s13014-026-02800-5

**Published:** 2026-02-12

**Authors:** Ang Qu, Lei Zhu, Weiqi Xiong, Ping Jiang, Hang Yang, Weijuan Jiang, Xiuwen Deng, Mengying Yang, Yanye Lu, Junjie Wang

**Affiliations:** 1 Department of Radiation Oncology, Peking University Third Hospital, 49 North Garden Road, Haidian District, Beijing, 100191 China; 2 Department of Biomedical Engineering, College of Future Technology, Peking University, Beijing, China; 3 Shanghai United Imaging Healthcare Co., Ltd., RT-IPH, Shanghai, China; 4 United Imaging Research Institute of Intelligent Imaging, CRI-BJ-IRTL, Beijing, China; 5 Institute of Medical Technology, Peking University Health Science Center, Peking University, No. 5 Yiheyuan Road, Haidian District, Beijing, 100871 China

**Keywords:** Endometrial cancer, Postoperative pelvic radiotherapy, Automatic delineation, Deep learning, Clinical target volume

## Abstract

**Background:**

The accurate delineation of the clinical target volume (CTV) is a critical step in precision radiotherapy for endometrial cancer (EC). Multi-phase CT provides more information for delineating the CTV. Our study aims to establish an innovative method for the specific delineation of CTV using multi-phase CT.

**Methods:**

Our multi-phase delineation datasets comprise 175 patients who received postoperative pelvic radiotherapy. These datasets include images of contrast-enhanced computed tomography (CECT) and non-contrast-enhanced computed tomography (NECT). Additionally, we introduce a novel framework for automatically segmenting the CTV using a deep learning model. The key component of our framework is the NCLNet, which fuses features from NECT and CECT within a Lightweight Network structure. This structure is optimized using a boundary-aware multi-phase learning strategy that we propose. In addition to the dice similarity coefficient (DSC) and the average symmetric surface distance (ASSD), we propose a novel contour dice similarity coefficient (CDSC) metric to evaluate the accuracy of the predictive outer contour. Three physicians modified the predictive CTV to assess the clinical utility of the proposed method.

**Results:**

The NCLNet achieves a DSC of 0.871 ± 0.027 and an ASSD of 0.878 ± 0.265 mm, with lower computation complexity (5.7 M parameters and FLOPS was 639.1G). In comparison, the widely used nnUNet attains a DSC of 0.860 ± 0.027 and an ASSD of 0.920 ± 0.286 mm, while requiring significantly more parameters (31.0 M) and similar FLOPs (643.8G). The overall evaluation against several benchmarks demonstrates better or comparable performance relative to methods with higher complexity. The average modification time and the modification volume percentage of the automatically delineated CTV were only 2.9 min and 3.61%, respectively. The CDSC of 8 mm thickness was 0.853 ± 0.030, demonstrating higher correlation with the clinical modification time of experts than both DSC and ASSD.

**Conclusions:**

The NCLNet generates high-quality automatic delineation of the CTV for postoperative pelvic radiotherapy in patients with EC.

## Introduction

Postoperative pelvic radiotherapy is an effective adjuvant therapy for endometrial cancer (EC). At present, intensity-modulated radiation therapy (IMRT) is recommended for radiation therapy [1]. The accurate and rapid delineation of clinical target volume (CTV) is important, ensuring the precise implementation of IMRT. The delineation of the CTV for postoperative adjuvant radiotherapy in EC was carried out according to the consensus guidelines published by RTOG in 2008 and 2020, utilizing anatomical landmarks [2, 3]. The CTVs are still manually delineated by radiation oncologists, mainly lasting for more than one hour for one patient. The accuracy of delineation is limited by doctors’ understanding of clinical consensus and experience, and there are differences in delineation quality, efficiency, and repeatability among radiation oncologists.

Deep learning-based methods have been widely applied in medical image delineation tasks in recent years [4]. Image segmentation based on convolutional neural networks (CNNs) can assist physicians to delineate CTV and organs at risk (OARs), exhibiting promising performance in organ delineation tasks. This significantly reduces the repetitive workload of physicians. Several studies have shown that deep learning-based delineation of OARs aligns well with clinical requirements, with small modifications required [5–11]. However, delineation of the CTV in radiotherapy is still a hard task because of its complex shapes, unclear boundaries, and inconsistent specification among physicians in different centers. Several studies have concentrated on the automatic delineation of CTV using deep learning-based methods for cervical cancer, prostate cancer, breast cancer, non-small cell lung cancer, and other brain and liver tumors [12–21]. For the automatic delineation of CTV in postoperative radiotherapy of EC, Kim et al. [22] evaluated the atlas-based algorithm, which was found to be time-consuming for the automatic delineation. No relevant deep learning-based research has yet been conducted, indicating the necessity of the development of accurate automated delineation methods.

Although contrast-enhanced computed tomography (CECT) demonstrates better sensitivity in delineating vascular boundaries than non-contrast-enhanced computed tomography (NECT), -CECT affects radiotherapy dose calculations due to the introduction of high-atomic-number contrast agents, and the effect on dose calculation remained controversial across studies [23–26]. The use of NECT was consistent with the patient’s treatment status. So, we usually developed radiotherapy plannings on NECT using CECT as the reference.

For this purpose, our work collects the multi-phase dataset in postoperative radiotherapy of EC to enable training a delineation model with multi-phase inputs for discerning CTV. However, traditional biological image delineation structures, e.g., UNet [27], nnUNet [28], struggle with fusing the multi-phase knowledge. Though Zhang et al. [29] proposed a mutual learning strategy for effective and robust cross-modality learning by fusing feature maps from two separable backbones, this approach suffers from high computational cost, limiting its clinical applicability.

In the present study, a multi-phase delineation framework is first proposed to automatically segment CTV in postoperative radiotherapy of EC using a lightweight two-branch network that effectively fuses the characteristics of NECT and CECT. Specifically, one branch is dedicated to the NECT modality (Branch-N), aiding in locating the CTV region for postoperative radiotherapy of EC. Meanwhile, the other branch (Branch-C) utilizes CECT, known for its specificity in delineating CTV boundaries, to enhance boundary adherence. The features extracted from these two branches are then fused and optimized using a proposed boundary-aware multi-phase learning (BAML) strategy to improve delineation performance. To address computational efficiency, a lightweight U-shaped extractor is introduced for both Branch-C and Branch-N, reducing the parameter size by 11 times compared to the original MAML [30]. This optimization aims to enhance the efficiency of handling multi-phase inputs. Experimental results indicate that our method outperforms existing approaches for CTV delineation in postoperative radiotherapy for EC, with significantly reduced computational complexity.

## Materials and methods

### Datasets

In the present study, considering the challenges posed by the blurriness of CTV boundaries, the nuanced differences in manual delineation, and the intricate correlation between boundaries and blood vessels, we attempted to initially assess the contribution of multi-phase CT to CTV delineation accuracy. To achieve this, we constructed the first multi-phase CT datasets for patients with endometrial cancer (EC).

Specifically, a total of 175 eligible cases of postoperative pelvic radiotherapy for EC who were admitted to the Peking University Third Hospital (Beijing, China) from 2014 to 2020 were enrolled. All patients were in a supine position, were fixed with a thermoplastic film. Images of NECT and arterial phase CECT with slice thickness of 5 millimeters were collected, and there were no metal artifacts in pelvic region. Images were acquired using a Brilliance CT Big Bore system (Philips Healthcare, Best, the Netherlands).

Statistically, the dataset comprises a population ranging from 24 to 78 years of age, with image voxel spacing from 0.736 to 1.365 mm in axial direction. The CTVs delineated on the NECT, with reference to the CECT, met the RTOG consensus and were uniformly reviewed and confirmed by an expert radiation oncologist, which were regarded as the ground truth. The delineated labels have lengths in the superior-inferior direction ranging from 15.5 to 23.0 cm, and the volume ranges from 375.1 to 995.0 cm^3^. Notably, the initial 142 cases were randomly assigned into training dataset (*n* = 114) and validation dataset (*n* = 28), while an additional 33 independently collected cases served as a test dataset. The study was approved by the ethics committee of Peking University Third Hospital.

### NCLNet structure

#### Overview

**Fig. 1:**
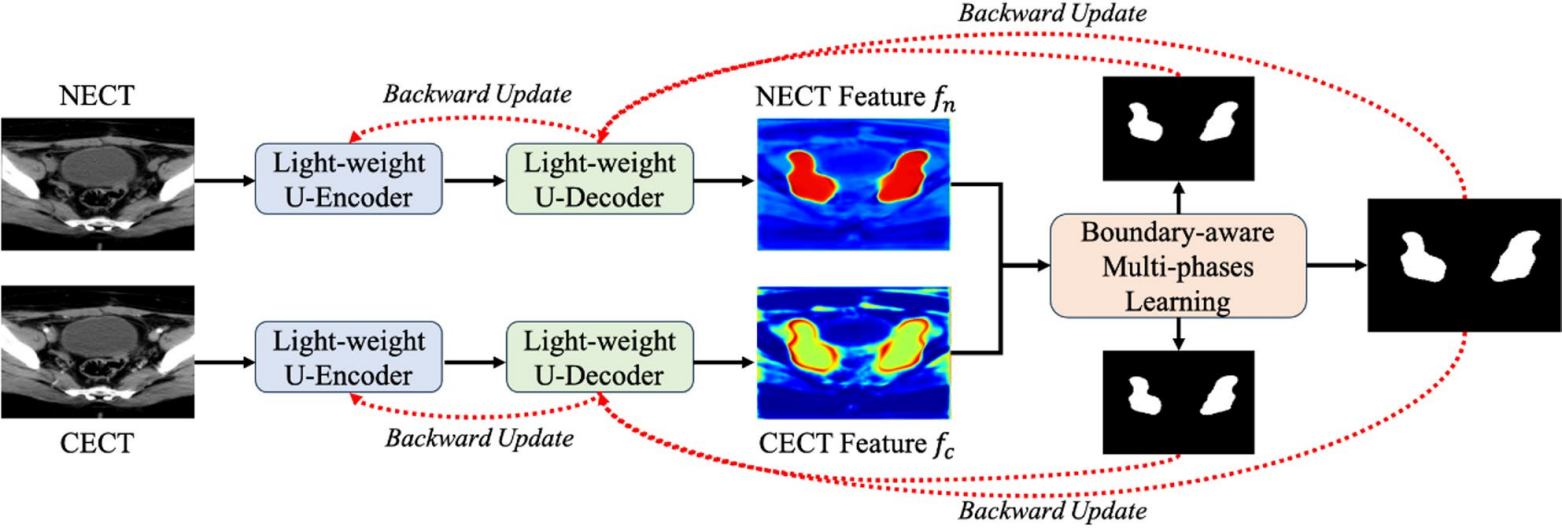
Overview of our method. It involves establishing the first multi-phase dataset and designing a multi-phase architecture for CTV segmentation in postoperative radiotherapy of EC

In the CTV delineation for EC, CECT is primarily used to help radiation oncologists identify key vascular structures. Specifically, CECT enables clear visualization of the iliac vessels, which are critical for defining the CTV contour. According to clinical guidelines, the CTV must include these vessels with a specified margin to ensure adequate treatment coverage. The CT images were categorized into three datasets: Dataset 1 containing only NECT images (NECT), Dataset 2 containing only CECT images (CECT), and Dataset 3 combining both NECT and CECT images (NEnCE). This categorization aimed to evaluate the impact of multi-phase CT on deep learning-based CTV segmentation.

In light of this thought, our framework utilizes an additional CECT modality to assist in delineation using NECT, viewing CTV delineation as a multi-phase segmentation task. As illustrated in Fig. 1, our NCLNet has a two-branch feature extractor, where Branch-N processes the NECT to generate features for locating the CTV ($\:{f}_{n})$, and Branch-C utilizes CECT to distinguish features around the CTV’s boundary ($\:{f}_{c}$). These two extractors are implemented using a proposed lightweight U-shape structure, employing bottleneck blocks to reduce computational cost. Furthermore, a boundary-aware multi-phase learning strategy transfers knowledge between modalities, enabling the network to capture more accurate CTV boundaries.

#### Lightweight U-shape extractor

**Fig. 2:**
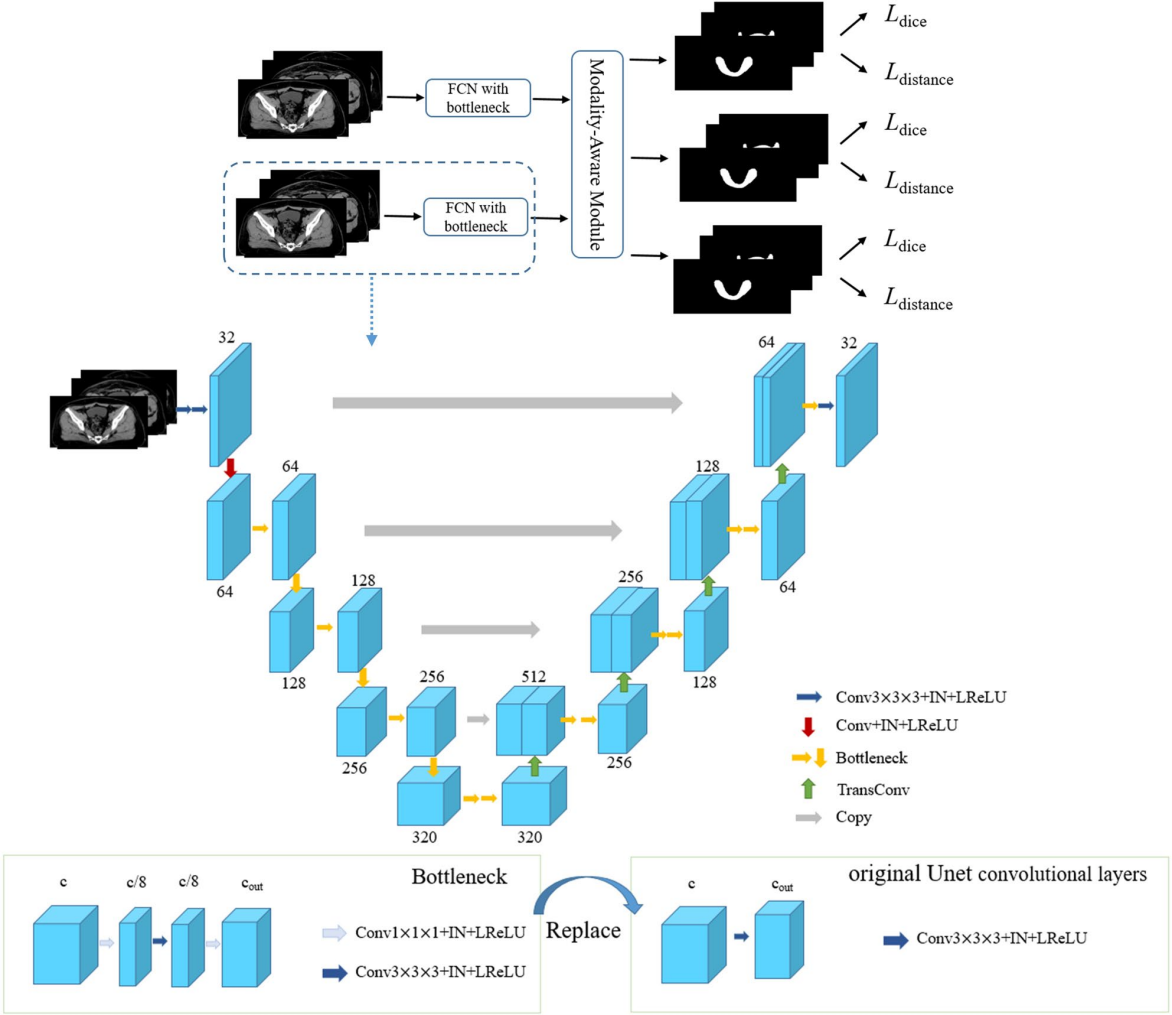
The structure of NCLNet, where original Unet convolutional layers are replaced with bottleneck blocks

Introducing an additional modality for segmentation requires the incorporation of an extra Fully Convolutional Network (FCN) for feature extraction, leading to an increase in the total number of model parameters. Unlike previous methods that simply halve the number of feature channels for each convolutional block [29], our framework addresses this challenge by introducing bottleneck structures to replace the original convolutional blocks. This innovative approach efficiently reduces computational costs without compromising the representation capacity.

As illustrated in Fig. 2, the proposed lightweight extractor employs a U-shaped structure. The bottleneck structures are positioned at the deep convolutional layers where the number of channels exceeds 32 to significantly reduce computational complexity. Specifically, these bottleneck blocks replace the original Unet convolutional layers with a three-layers configuration comprising: an initial $\:1\times\:1\times\:1$ convolution for channel compression, followed by a $\:3\times\:3\times\:3$ convolution, and finally another $\:1\times\:1\times\:1$ convolution for channel restoration.

Let $\:c$ and $\:{c}_{out}$ denote the numbers of input and output channels. The number of parameters of the bottleneck structure is formulated as follows:

$$\begin{aligned}&\:c\times\:\frac{c}{8}\times\:1\times\:1\times\:1+\frac{c}{8}\times\:\frac{c}{8}\times\:3\times\:3\times\:3\cr&+\frac{c}{8}\times\:{c}_{out}\times\:1\times\:1\times\:1\end{aligned}$$

This results in a reduction of approximately 40 times when $\:{c}_{out}$ equals to $\:c$, compared with the original number of parameters $\:c\times\:{c}_{out}\times\:3\times\:3\times\:3$ in a normal convolutional kernel. By replacing all the convolutional layers with a bottleneck structure of each modality’s FCN, the number of network parameters are significantly reduced.

#### Boundary-aware multi-phase learning strategy

As mentioned, multi-phase CT provide more information assists in delineating the boundary of CTV. To enhance contour accuracy, our framework incorporates a proposed boundary-aware multi-phase learning strategy. This strategy takes into consideration the weight of the outer contour when fusing the multi-phase features.

As depicted in Fig. 2, after extracting the modality-modality features with the two lightweight U-shape extractors, the multi-aware module [29] is employed to fuse the features of Branch-C and Branch-N to produce three segmentation maps:

1 $$\:{p}_{ce}=conv\left({f}_{c}\right)$$

2 $$\:{p}_{ne}=conv\left({f}_{n}\right)$$

3 $$\:{p}_{fuse}=m({f}_{n},\:{f}_{c})$$

Here, $\:{p}_{ce}$, $\:{p}_{ne}$, $\:{p}_{fuse}$ represent the predictions with CECT features, NECT features and fusion features, respectively. $\:conv$ represents the convolutional operation and $\:m(\cdot,\cdot\:)$ represents the multi-aware module operation.

Subsequently, we apply both Dice coefficient loss and distance penalty [31, 32] as loss functions to supervise the three segmentation maps to facilitate knowledge transferring, where the distance penalty helps to fully use the boundary information provided by CECT modality. Let $\:F$ be the foreground of $\:G$, where $\:{\forall\:y}_{i}\in\:F,\:{q}_{i}=1$ and $\:{\forall\:y}_{i}\notin\:F,\:{q}_{i}=0$. And let $\:B$ be the background of $\:G$, which is defined as $\:B=G-F$. The distance transform fields of *F* and *B* compute the minimum Euclidean distance from each voxel to the background and foreground:

4 $$\:{D\left(F\right)}_{i}=\:\left\{\begin{array}{c}\:\underset{{b}_{j}\in\:B}{\mathrm{min}}\left\{{\parallel{x}_{i}-{b}_{j}\parallel}_{2}\right\}\:\:\:\:for\:{x}_{i}\in\:F\\\:\:\:\:\:\:\:0\:\:\:\:\:\:\:\:\:\:\:\:for\:{x}_{i}\in\:B\end{array}\right.$$

5 $$\:{D\left(B\right)}_{i}=\:\left\{\begin{array}{c}\:\:\:\:\:\:0\:\:\:\:\:\:\:\:\:\:\:\:for\:{x}_{i}\in\:F\\\:\:\underset{{f}_{j}\in\:F}{\mathrm{min}}\left\{{\parallel{x}_{i}-{f}_{j}\parallel}_{2}\right\}\:\:\:\:for\:{x}_{i}\in\:B\end{array}\right.$$

Then, the distance to the outer contour of all voxels via a combination of $\:{D\left(F\right)}_{i}$ and $\:{D\left(B\right)}_{i}\:$can be computed. We then reverse and normalize these distance fields to construct a contour refinement penalty, where the distance penalty weight matrix $\:w$ was formulated as follows:

6 $$\begin{aligned}\:{w}_{i}=&\:\frac{\underset{1\le\:i\le\:N}{\mathrm{max}}\left\{{D\left(F\right)}_{i}\right\}-{D\left(F\right)}_{i}}{\underset{1\le\:j\le\:N}{\mathrm{max}}\left\{\underset{1\le\:i\le\:N}{\mathrm{max}}\left\{{D\left(F\right)}_{i}\right\}-{D\left(F\right)}_{j}\right\}}\:\cr&+\:\frac{{\underset{1\le\:i\le\:N}{\mathrm{max}}\left\{{D\left(B\right)}_{i}\right\}-D\left(B\right)}_{i}}{\underset{1\le\:j\le\:N}{\mathrm{max}}\left\{{\underset{1\le\:i\le\:N}{\mathrm{max}}\left\{{D\left(B\right)}_{i}\right\}-D\left(B\right)}_{j}\right\}}\end{aligned}$$

The final loss function $\:L$ is the summation of distance penalty dice and binary cross-entropy loss function.

7 $$\begin{aligned}\:L\:&=\:\frac{2\sum\:_{i=1}^{N}{w}_{i}{p}_{i}{q}_{i}}{\sum\:_{i=i}^{N}{p}_{i}+\sum\:_{i=i}^{N}{q}_{i}}\cr&+(-\frac{1}{N}\sum\:_{i=1}^{N}{(q}_{i}\mathrm{log}{p}_{i}+{(1-q}_{i})\mathrm{log}{(1-p}_{i}\left)\right))\end{aligned}$$

#### Implementation details

During the data preprocessing phase, images were first normalized by truncating intensity values to the 0.5th and 99.5th percentiles of the Hounsfield unit (HU) distribution. Subsequently, images were resampled to a uniform voxel size of [5 mm, 1 mm, 1 mm] based on the median voxel spacing observed in the training dataset. For data augmentation stage, random flipping (along the *x*, *y*, and *z* axes) was probabilistically applied to each image, followed by random sampled rotation angles (− 15° to + 15°) around each axis. Finally, all images were cropped to a consistent size of (40, 224, 192) voxels. For the NCLNet architecture, the number of features for the five downsampling layers were set to 32, 64, 128, 256, and 320 respectively, with the upsampling layers mirroring these values. The SGD optimizer with a momentum of 0.95 was used, and the learning rate was initially set to 0.01, decaying linearly to zero over the training epochs. The mini-batch size was set to 2. Training was conducted for 200 epochs, with each epoch consisting of 250 iterations. Kaiming Initialization [33] was utilized, and the training was performed using PyTorch on a single NVIDIA Quadro RTX 4000 GPU and two Intel Xeon Gold 5128 CPUs.

### Evaluation

#### Evaluation metrics

The agreement between the prediction (automatic delineation by our NCLNet framework) and the ground truth of CTV was quantitatively evaluated by various overlap and distance metrics. The Dice Similarity Coefficient (DSC) is widely accepted as a standard measure for the spatial overlap between the volumetric masks of prediction and ground truth. The Average Symmetric Surface Distance (ASSD) was also utilized as an evaluation metric, quantifying the average Euclidean distance between corresponding surface points of the predicted and ground truth masks. Following the definition in Sect. “Boundary-aware multi-phase learning strategy”, the DSC and ASSD metrics were defined as follows:

8 $$\:DSC=\:\frac{2\sum\:_{i=1}^{N}{p}_{i}{q}_{i}}{\sum\:_{i=i}^{N}{p}_{i}+\sum\:_{i=i}^{N}{q}_{i}}$$

9 $$\:ASD\left(P,G\right)=\:\frac{\sum\:_{{x}_{i}\in\:{F}_{P}}\underset{{y}_{j}\in\:{F}_{G}}{\mathrm{min}}\left\{{\parallel{x}_{i}-{y}_{j}\parallel}_{2}\right\}}{\sum\:_{i=i}^{N}{p}_{i}}$$

10 $$\:ASSD\left(P,G\right)=\frac{ASD\left(P,G\right)+ASD(G,P)}{2}$$

Here, $\:{F}_{P}$ and $\:{F}_{G}$ represent the foreground of $\:P$ and $\:G$, respectively.

**Fig. 3:**
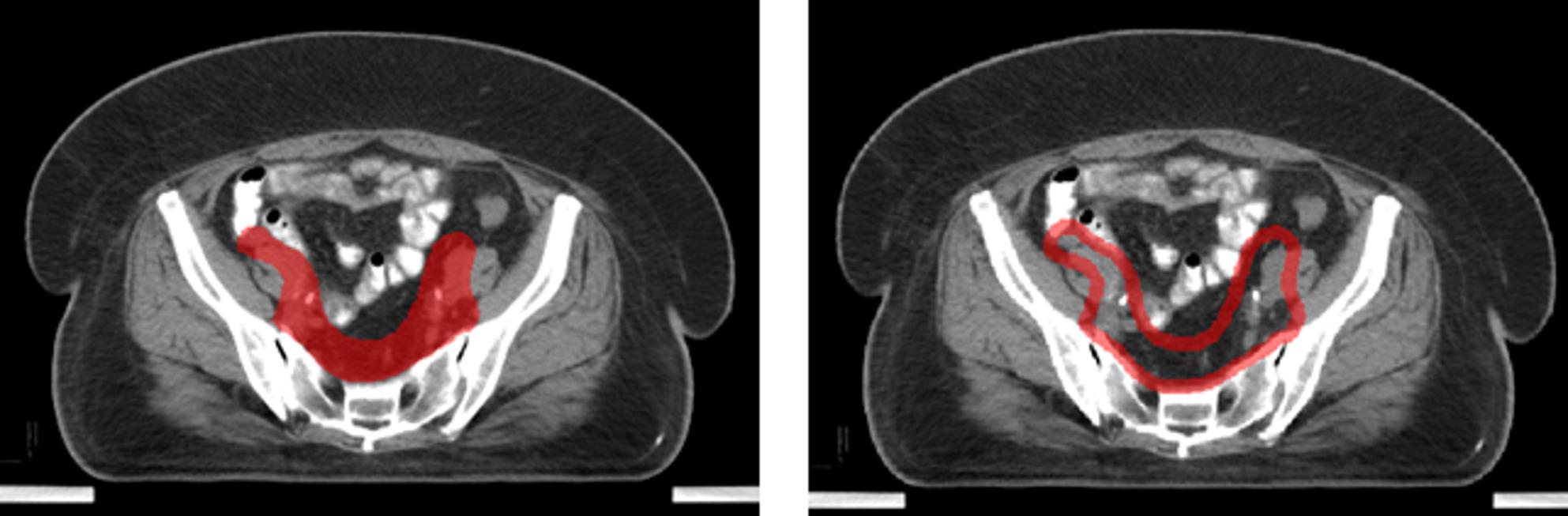
The mask and the generated outer contour with an 8 mm thickness

Clinically, the primary discrepancies in delineation arise from the boundaries of the CTV. Thus, in addition to the geometric evaluation metrics for segmentation (DSC, ASDD), we proposed a Contour Dice Similarity Coefficient (CDSC) metric that measures the overlap between the outer contours of the masks. Specifically, masks of varying thicknesses were generated along the outer contours at each slice from the prediction and ground truth masks using the distance fields. The 2D DSC of these outer contour masks was subsequently calculated and the average across slices was obtained. CDSC reflects the accuracy of the outer contour of the predictive mask. Figure 3 is an example of a mask and the corresponding outer contour with an 8 mm thickness.

#### Clinical evaluation

The predicted CTVs of the test dataset were reviewed and modified by three physicians with different qualifications, including one attending physician, one chief physician, and one radiation oncologist with more than 20 years of experience. Each clinician independently assessed the predictions without access to the ground truth or others’ modifications. The recorded modification time for each case was analyzed, and the pre- and post-modified CTVs, the post-modified and the ground truth of CTVs were compared using geometric similarity metrics respectively.

### Statistical analysis

In the statistical analysis, evaluation metrics were expressed as mean ± standard deviation (SD) and were compared between different methods using paired two-tailed t-tests. The statistical analysis was performed using SPSS 24.0 software (IBM, Armonk, NY, USA). A P-value less than 0.05 was indicative of statistical significance.

## Results

### Effectiveness of multi-phase CT

**Fig. 4:**
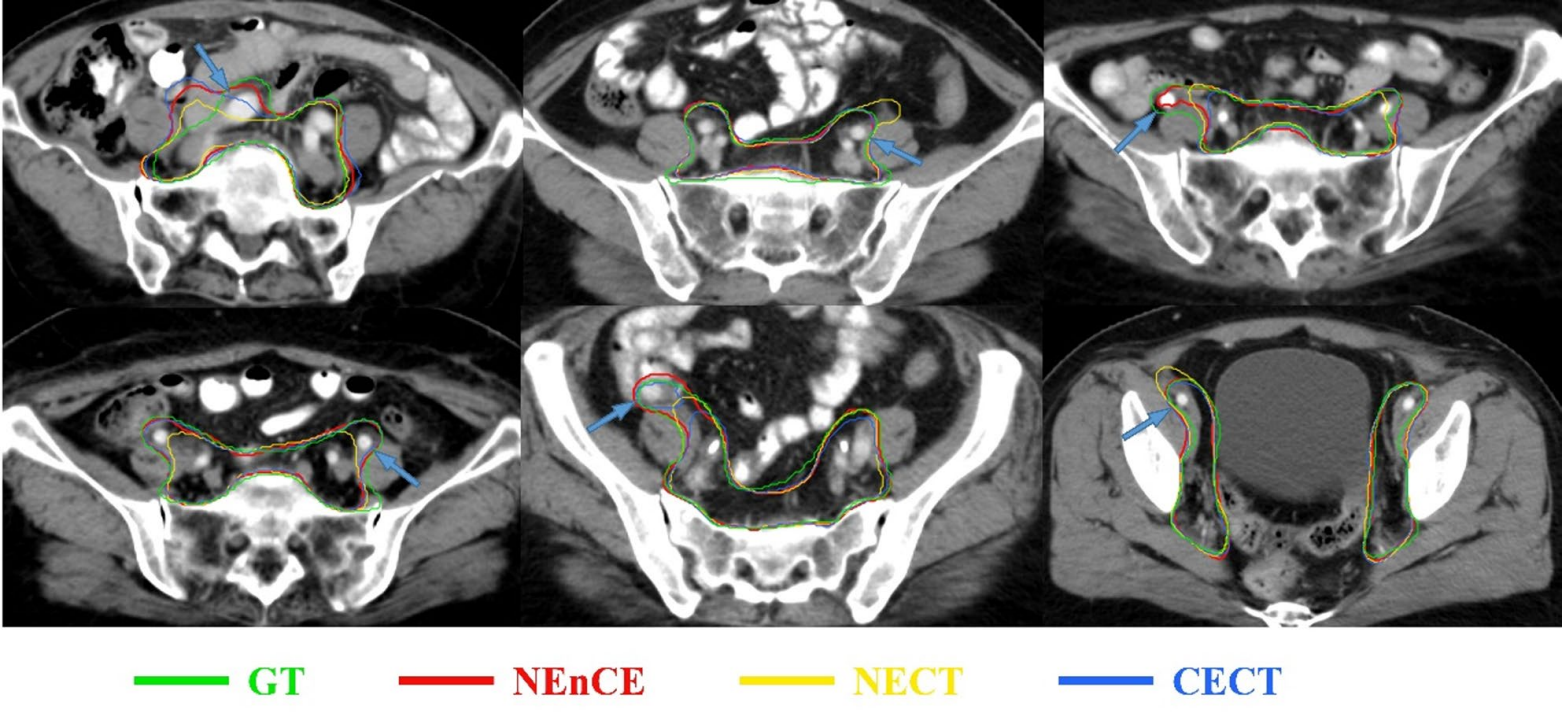
CTV contours are depicted on CECT images of 6 cases. The iliac artery is indicated by the blue arrow. All images have a window level of 50 HU and a window width of 300 HU. GT was ground truth, as manually delineation of CTV; NEnCE was automatic delineation of CTV by our NCLNet framework via multi-phase CT; NECT was automatic delineation of CTV by our NCLNet framework via only NECT; CECT was automatic delineation of CTV by our NCLNet framework via only CECT

Our framework incorporates an additional CECT modality that specializes in delineating the boundary of CTV to assist in the delineation of patients with endometrial cancer, by viewing it as a multi-phase segmentation task. In addition to our method with 2D and 3D backbone structures, we evaluated six methods [28, 29, 34–37] including the basic network and innovative structures previously proposed for CTV automatic delineation in literature, under single-phase (NECT or CECT) and multi-phase inputs to demonstrate the effectiveness of utilizing multi-phase CT. The other six methods, which uses NEnCE, fuses images by concatenating them along the channel dimension—an early fusion strategy. This allows the CNN to automatically learn how to combine the two modalities from its first layer, eliminating the need for predefined fusion rules. As illustrated in Table 1, most methods trained with NEnCE achieved a higher DSC and a lower ASSD than those trained on NECT or CECT. Our method, the standard version (two-extractor configuration) and single-extractor configuration version are shown in Table 2. The results of CECT consistently outperformed those of NECT, except in the case of the standard version of NCLNET-2D (two-extractor configuration), where the differences were not statistically significant. Notably, NEnCE demonstrated optimal performance, achieving the highest DSC and lowest ASSD. Figure 4 presents the slices of CECT images along with contours of the ground truth, NECT-based predictions, and NEnCE-based predictions for six cases. When focusing on the region surrounding the iliac arteries, the NEnCE-based prediction (depicted in red) closely aligns with the ground truth (displayed in green).

**Table 1:** Evaluation of the CTV in different datasets

Method	Metrics	NECT	CECT	NEnCE	*P*_1_	*P*_2_	*P*_3_
RA-CTVNet [35]	DSC	0.763 ± 0.095	0.730 ± 0.090	0.760 ± 0.084	0.000	0.598	0.000
	ASSD (mm)	2.572 ± 1.088	2.884 ± 1.184	3.044 ± 1.042	0.000	0.002	0.013
DpnUNet [34]	DSC	0.803 ± 0.032	0.828 ± 0.036	0.831 ± 0.026	0.000	0.000	0.415
	ASSD (mm)	1.466 ± 0.340	1.149 ± 0.337	1.166 ± 0.285	0.000	0.000	0.623
nnFormer [36]	DSC	0.867 ± 0.027	0.873 ± 0.023	0.868 ± 0.029	0.051	0.672	0.090
	ASSD (mm)	0.996 ± 0.228	0.966 ± 0.224	1.001 ± 0.270	0.228	0.847	0.087
STUNet [37]	DSC	0.863 ± 0.028	0.869 ± 0.029	0.868 ± 0.028	0.020	0.091	0.552
	ASSD (mm)	1.048 ± 0.283	0.986 ± 0.269	1.220 ± 0.283	0.071	0.042	0.402
nnUNet [28]	DSC	0.851 ± 0.026	0.856 ± 0.026	0.860 ± 0.027	0.012	0.000	0.028
	ASSD (mm)	1.037 ± 0.289	0.973 ± 0.282	0.920 ± 0.286	0.075	0.001	0.017
MAML [29]	DSC	0.851 ± 0.023	0.858 ± 0.023	0.865 ± 0.027	0.000	0.000	0.000
	ASSD (mm)	1.054 ± 0.248	0.968 ± 0.225	0.927 ± 0.263	0.000	0.000	0.025

Note: $\:{P}_{1}$-value denotes the comparison between CECT and NECT, $\:{P}_{2}$-value denotes the comparison between NEnCE and NECT, and $\:{P}_{3}$-value denotes the comparison between NEnCE and CECT

**Table 2:** Evaluation of the CTV for NCLNet

Method	Metrics	NECT	CECT	NEnCE	*P*_1_	*P*_2_	*P*_3_
NCLNet − 2D	DSC	0.847 ± 0.025	0.848 ± 0.024	0.863 ± 0.300	0.516	0.001	0.002
	ASSD (mm)	1.107 ± 0.301	1.092 ± 0.308	0.908 ± 0.236	0.704	0.000	0.001
NCLNet − 2D (single-extractor)	DSC	0.842 ± 0.033	0.855 ± 0.027	-	0.001	0.000	0.047
	ASSD (mm)	1.206 ± 0.470	1.053 ± 0.337	-	0.048	0.001	0.005
NCLNet − 3D	DSC	0.853 ± 0.027	0.857 ± 0.025	0.871 ± 0.027	0.022	0.000	0.000
	ASSD (mm)	1.106 ± 0.312	1.049 ± 0.303	0.878 ± 0.265	0.016	0.000	0.001
NCLNet − 3D (single-extractor)	DSC	0.853 ± 0.029	0.864 ± 0.031	-	0.041	0.000	0.038
	ASSD (mm)	1.285 ± 0.415	1.114 ± 0.454	-	0.048	0.000	0.000

Note: $\:{P}_{1}$-value denotes the comparison between CECT and NECT, $\:{P}_{2}$-value denotes the comparison between NEnCE and NECT, and $\:{P}_{3}$-value denotes the comparison between NEnCE and CECT. For single-extractor NCLNet models: $\:{P}_{2}$ and $\:{P}_{3}$ values are calculated between two-extractor configuration (NEnCE) and single-extractor configurations (NECT and CECT)

### Effectiveness of NCLNet structure

We compared the proposed NCLNet with DpnUNet [34], RA-CTVNet [35], nnFormer [36], STU-Net [37], nnUNet [28] and MAML [29] in terms of performance on multimodal segmentation. RA-CTVNet and DpnUNet are target segmentation models used in radiotherapy for cervical cancer, which are similar to our task. nnFormer is based on a transformer architecture, while STU-Net enhances the standard convolutional blocks employed in nnUNet. nnUNet and MAML serve as our baseline methods, having achieved good performance in medical image segmentation and multi-modal segmentation, respectively. All networks were trained to convergence, and the best model parameters were selected.

**Fig. 5:**
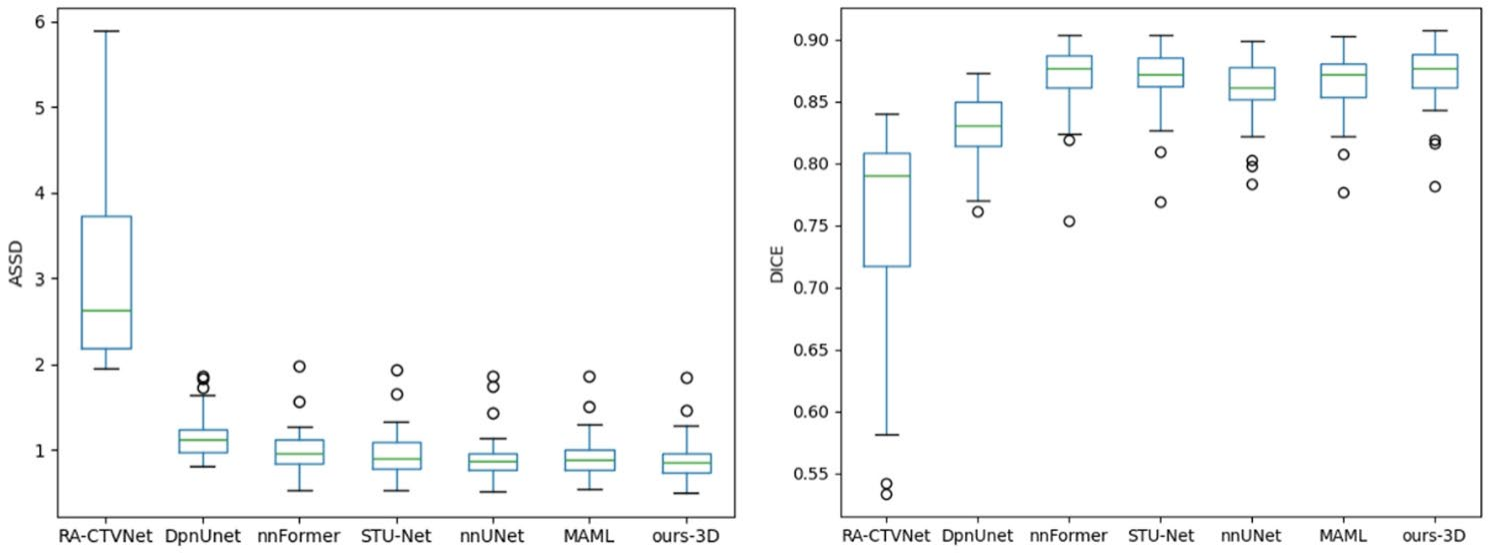
DSC and ASSD of different methods with 3D inputs

**Fig. 6:**
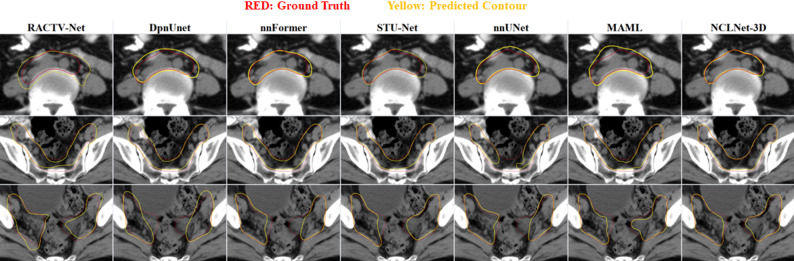
The comparison of different methods and baselines on CTV delineation (NEnCE)

In terms of indicated in Table 3, combining performance metrics and parameter size, NCLNet-3D achieved the best performance with the fewest parameters. Moreover, due to the bottleneck blocks, the number of parameters of NCLNet is only 9.2% of that of MAML and the floating point operations per second (FLOPS) is 38.3% of that of MAML. It should be noted that DpnUNet is a 2.5D network. NCLNet-3D outperformed most of other models in both metrics. For the 3D inputs, it showed slightly better mean values than nnFormer and STU-Net on DSC and nnUNet on ASSD, although the differences were not statistically significant. For 2D inputs, our method also adapted effectively, although a few models had numerically higher metric means without statistical significance. While Unet and SwinUNetR had fewer parameters than NCLNet-2D, their DSC and ASSD performance was inferior. Figure 5 also illustrates a box plot displaying the results for 3D inputs models. Additionally, Fig. 6 visualizes the CTV contours and differences among these methods.

**Table 3:** Evaluation of the CTV predicted by different methods

Method	DSC		ASSD		Parameters	FLOPS
	Mean ± SD	*P*	Mean ± SD	*P*		
RA-CTVNet [35]	0.760 ± 0.083	0.000	3.044 ± 1.042	0.000	44.8 M	790.6G
DpnUNet-2.5D [34]	0.831 ± 0.026	0.000	1.166 ± 0.285	0.000	126.6 M	73.8G
nnFormer [36]	0.868 ± 0.029	0.228	1.001 ± 0.270	0.000	37.5 M	271.6G
STU-Net [37]	0.868 ± 0.028	0.200	1.220 ± 0.283	0.001	58.3 M	510.0G
nnUNet [28]	0.860 ± 0.027	0.000	0.920 ± 0.286	0.056	31.0 M	643.8G
MAML [29]	0.865 ± 0.027	0.001	0.927 ± 0.263	0.010	62.2 M	1668.1G
**NCLNet-3D**	**0.871 ± 0.027**	**-**	**0.878 ± 0.265**	**-**	**5.7M**	**639.1G**
UNet [27]	0.808 ± 0.075	0.023	4.937 ± 8.851	0.042	1.63 M	4.195G
SwinUNetR [38]	0.825 ± 0.074	0.004	3.799 ± 5.948	0.006	6.28 M	16.65G
UNet++ [39]	0.826 ± 0.088	0.085	1.425 ± 1.051	0.116	9.163 M	122.53G
AttUNet [40]	0.830 ± 0.083	0.004	1.303 ± 0.672	0.121	34.88 M	223.91G
**NCLNet-2D**	**0.863 ± 0.030**	**-**	**0.908 ± 0.236**	-	8.12 M	21.07G

### Ablation study

We conducted an ablation study to systematically evaluate the contributions of the bottleneck (BN) lightweight U-shaped encoder and the distance penalty (DP) loss function in our proposed NCLNet. As shown in Table 4, the baseline model without these components achieved a DSC of 0.865 ± 0.027 and an ASSD of 0.927 ± 0.263 mm. Notably, integrating the DP loss improved performance primarily in ASSD, while the training time (per epoch) increased significantly, almost doubling the baseline’s training time. When using the BN component alone, it resulted in decreased performance in both DSC and ASSD, while significantly decreasing the training time (per epoch) compared to the baseline. The optimal configuration combined both BN and DP, yielding improvements in both metrics and reducing computational requirements, with the training time increasing by 6.1 min relative to the baseline but decreasing by 5.8 min compared to using DP alone. The model enhanced inference accuracy, reduced parameters, and avoided a significant increase in training time (per epoch), and reducing the mean inference time per case to 31.0s.

**Table 4:** Evaluation of the CTV predicted by different methods

	DSC	*P*-value	ASSD (mm)	*P*-value	Training time	Inference time	Parameters	FLOPS
Baseline	0.865 ± 0.027	-	0.927 ± 0.263	-	12.0 min	33.6s	62.2 M	1668.1G
+ DP	0.868 ± 0.024	0.229	0.874 ± 0.250	0.023	23.9 min	33.5s	62.2 M	1668.1G
+ BN	0.861 ± 0.024	0.009	0.978 ± 0.240	0.001	7.4 min	31.2s	5.7 M	639.1G
+ BN&DP	0.871 ± 0.027	0.001	0.878 ± 0.265	0.010	18.1 min	31.0s	5.7 M	639.1G

### Clinical evaluation

Three radiation oncologists modified the predicted CTVs of the proposed NCLNet, and the evaluation results are illustrated in Table 5. The prediction and modified CTVs were compared, and as shown in Table 5(a), the average values of DSC, ASSD, and modified percentage achieved by the three physicians were 0.982, 0.125 mm, and 3.61%, respectively. The average modified time was only 2.9 min. As presented in Table 5(b), the predicted and modified CTVs were compared with the ground truth. The DSC, ASSD, and CDSC of the post-modified CTVs showed limited improvement over the prediction. There was no statistically significant difference in the average results attained by the three radiation oncologists compared with those of the predicted CTVs ($\:P\ge\:0.05$).

**Table 5:** Evaluation of physicians’ modifications. PhyAve is the average of 3 physicians

**(a) Metrics of physicians’ modifications compared with the proposed prediction** ^*^							
	DSC	ASSD(mm)		Modified volume (mm^3^)		Modified percentage	Modified time (min)
Pred-Phy1	0.975±0.010	0.154±0.077		33.49±14.67		5.04±1.88%	2.02±1.15
Pred-Phy2	0.984±0.010	0.121±0.086		21.15±13.68		3.14±1.89%	3.82±1.93
Pred-Phy3	0.987±0.009	0.101±0.070		17.75±13.09		2.65±1.83%	2.68±0.88
Pred-PhyAve	0.982±0.008	0.125±0.064		24.13±11.56		3.61±1.51%	2.90±1.63

**(b) Metrics of physicians’ modifications compared with the ground truth** ^**^							
	DSC		ASSD (mm)			CDSC -8mm	
	Mean±SD	*p*-value	Mean±SD	*p*	Mean±SD	*p*	
GT-Pred	0.883±0.018	-	0.790±0.173		-	0.884±0.023	-
GT-Phy1	0.884±0.013	0.89	0.774±0.152		0.51	0.883±0.019	0.59
GT-Phy2	0.890±0.016	0.01	0.723±0.145		0.03	0.890±0.024	0.02
GT-Phy3	0.886±0.018	0.08	0.740±0.157		0.01	0.885±0.022	0.09
GT-PhyAve	0.886±0.016	0.07	0.746±0.147		0.05	0.887±0.021	0.06

Note: *, the proposed prediction was automatic delineation of CTVs by our NCLNet framework; **, manually delineation of CTVs and confirmed by an expert radiation oncologist

### The evaluation of CDSC

**Fig. 7:**
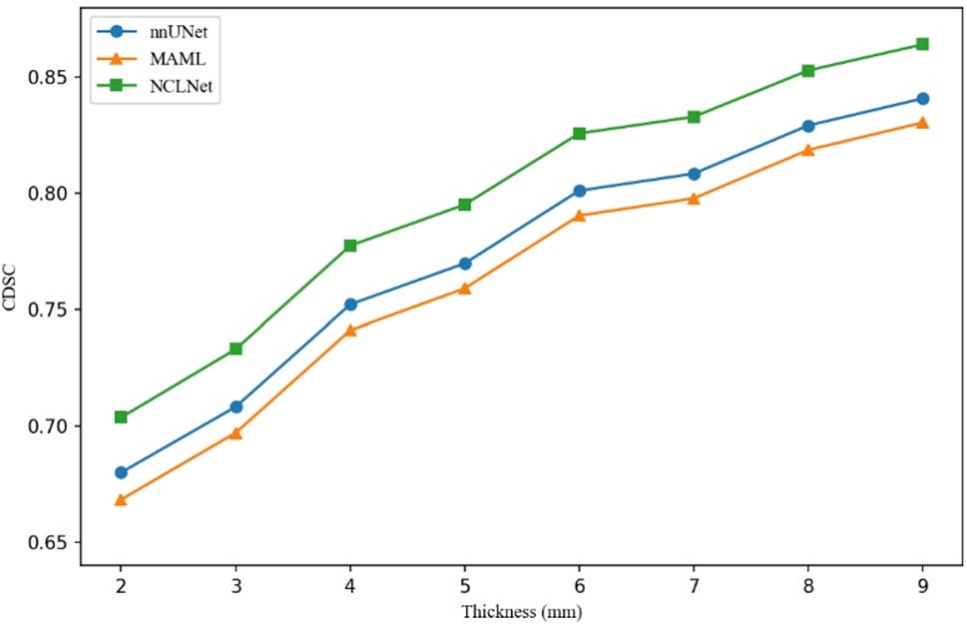
The CDSC for baseline methods and NCLNet

To further verify the accuracy of the method for outer contour prediction, we attempted to calculate the proposed CDSC across several thicknesses for the three methods. Figure 7 displays the average CDSC for our methods and baseline methods. A Thickness range of 2–9 mm was selected because CDSC did not significantly improve when the thickness was further increased. The proposed NCLNet achieved the highest CDSC across all thicknesses.

In addition, we illustrate the effectiveness of the CDSC metrics. Specifically, we calculated the Pearson correlation coefficients between each metric and the physicians’ clinical modification times. The correlation coefficients of DSC, CDSC of 8 mm and ASSD with the median modification time were − 0.48, -0.54 and 0.41, respectively. We selected 8 mm CDSC as the primary metric due to its strongest correlation with the median modification time compared to the other thickness. It is important to note that DSC and CDSC are negatively correlated with modification time, while ASSD exhibits a positive correlation. 8 mm CDSC demonstrates a higher absolute correlation coefficient compared to both DSC and ASSD.

## Discussion

In this study, a lightweight framework was proposed to automatically segment CTV in postoperative radiotherapy of endometrial cancer using multi-phase CT. The proposed NCLNet for CTV delineation achieved a DSC of 0.871 ± 0.027, an ASSD of 0.878 ± 0.265 mm, while demonstrating a significant reduction in computational complexity with only 5.7 million parameters and a mean inference time of 31.0 s per case.

Traditional manual contouring of target volumes is time-intensive and reliant on physicians’ expertise. Deep learning-based delineation presents a solution to enhance accuracy, productivity, and efficiency in medical practice. The utilization of artificial intelligence (AI) has reduced delineation time for primary tumor volumes or OARs by approximately 10–12 min [41, 42]. The CTV, defined as the subclinical tumor area beyond visible tumor boundaries, poses a challenging segmentation task, surpassing even the complexity of OAR and tumor delineation. Balagopal et al. [43] proposed the Anatomy Guided Multi-Task Network (AG-MTN) for a postoperative tumor bed in prostate cancer, which models physicians’ behavior by utilizing surrounding organs as anatomical landmarks. This method achieved an average DSC of 0.87 for CTV delineation. Monte-Carlo dropout (MCDO) was employed to estimate model uncertainty, aiding physicians in identifying areas requiring correction. Song et al. [44] developed the DeepLabv3 + network for semantic feature extraction in rectal CTV delineation, achieving a DSC of 0.88. Liu et al. [34] introduced DpnUNet for CTV segmentation in locally advanced cervical cancer, employing a 2.5D architecture that uses three adjacent CT slices as input to capture spatial context efficiently while maintaining computational efficiency. Additionally, RA-CTVNet [35], enhanced CTV delineation in cervical cancer CT images by incorporating area-aware reweighting and recursive refinement strategies. The area-aware reweighting strategy increased DSC by 3.3% on average, and the recursive refinement strategy further improved DSC by 1.6% on average, compared to the backbone network.

The postoperative pelvic radiotherapy CTV for EC typically encompasses draining lymphatics, the parametrium, and the upper vagina, mirroring the approach in cervical cancer, while exhibiting larger dimensions compared to other pelvic tumors. Anatomically, the CTV is divided into three superior segments [3]. However, these segments lack continuity and are significantly influenced by rectal and bladder filling, rendering automatic CTV delineation challenging. To Address this, Kim et al. [22] excluded the vaginal cuff CTV and focused on automated segmentation of the pelvic nodal CTV using an atlas-based algorithm, owing to substantial volume variations in the vaginal cuff.

Considering the challenges posed by the blurriness of CTV boundaries, the nuanced differences in manual delineation, and the intricate correlation between boundaries and blood vessels, this study initially aimed to assess the contribution of multi-phase CT to CTV delineation accuracy. We employed well-established networks (nnUNet and MAML), novel networks (nnFormer and STUNet), and two additional architectures (RA-CTVNet and DpnUNet) designed for cervical cancer. Most of method strained on CECT achieved a higher DSC and a lower ASSD compared with NECT, demonstrating that CECT improves CTV segmentation. Notably, most of the models trained on the NEnCE dataset predicted significantly more accurate CTV boundaries than those trained on NECT or CECT alone, indicating that multi-phase CT enhances target delineation accuracy. Inspired by the performance gains from multi-phase inputs, we developed a two-branch network optimized for segmentation with dual-phase inputs. MAML, designed for multi-modal segmentation by integrating cross-modal features, outperformed nnUNet in this context. However, increasing modalities substantially escalated network parameters. To address this, NCLNet was introduced, leveraging lightweight U-shaped extractors with only 1/11 the parameters of MAML. This design drastically reduced computational costs, memory usage, and enabled streamlined deployment. To tackle the challenge of predicting CTV surrounding iliac vessels, we further optimized NCLNet with a boundary-aware multi-phase learning strategy. This approach incorporated a distance penalty loss to refine outer contours, improving mean ASSD by 10.2%.

Overall, our method achieved a DSC of 0.871 ± 0.027 and an ASSD of 0.878 ± 0.265 mm with mean inference time 31.0s per case. NCLNet outperformed RA-CTVNet and DpnUNet (both specialized for cervical cancer CTV segmentation) on the same dataset. Compared to novel networks like nnFormer and STUNet, NCLNet achieved better ASSD while using substantially fewer parameters and lower FLOPs.

Clinically, the proposed CTV predictions required only minor modifications during evaluation by radiation oncologists. The average modification time was a mere 2.9 min, accounting for a modification percentage of 3.61%, indicating the high accuracy of the model. Although individual variations among doctors were observed, reflecting their preferences and experiences, no statistically significant differences were found in the average results achieved by the three oncologists compared to the pre-modification baseline (*P* ≥ 0.05). This robustly demonstrated the clinical feasibility of the proposed predictions for CTV delineation.

For specific assessment of outer contour prediction accuracy, the CDSC was used as an evaluation metric. The proposed method excelled, achieving the highest CDSC for thicknesses ranging from 2 to 9 mm, indicating optimal performance in outer contour prediction. Meanwhile, the 8 mm CDSC demonstrated higher correlation with the clinical modification time of oncologists than did the DSC and ASSD.

While the current study presented promising results, limitations are noteworthy. Notably, the validation of the proposed method was confined to a single-center dataset of EC patients undergoing radiotherapy, primarily due to dataset constraints. To enhance the robustness of the findings, efforts will be made to expand the dataset by collecting data from multiple institutions. This broader dataset will provide a more comprehensive evaluation of the proposed model’s performance across diverse patient populations.

## Conclusion

In conclusion, the innovative framework may serve as a valuable tool for physicians, facilitating the delineation of CTV in postoperative pelvic radiotherapy with high speed, consistency, efficiency, and precision. However, it is imperative to emphasize that the proposed method may assist radiation oncologists in the CTV delineation process. The complexity of individual cases demands the nuanced expertise and judgment of experienced professionals. Moreover, there are variations among institutions and physicians in their approaches to CTV delineation. Our future research will explore the potential and feasibility of adaptive models for multi-center applications.

## Data Availability

The datasets generated and analyzed during this study are not publicly available due to institutional data governance policies but are available from the corresponding author on reasonable request. Data sharing is subject to approval by the originating hospital and requires a formal data use agreement.

## Abbreviations

CTV: Clinical target volume
EC: Endometrial cancer
CECT: Contrast-enhanced computed tomography
NECT: Non-contrast-enhanced computed tomography
DSC: Dice similarity coefficient
ASSD: Average symmetric surface distance
CDSC: Contour dice similarity coefficient
